# Supplementary material for: All-at-once RNA folding with 3D motif prediction framed by evolutionary information
Source: Nat Methods. 2025 Oct 3;22(10):2094–106. doi: 10.1038/s41592-025-02833-w (PMC12510878; doi:10.1038/s41592-025-02833-w)
Supplement: Supplementary file 3 — Source code to run CaCoFold-R3D. Data and results. [file 41592_2025_2833_MOESM3_ESM.gz › supplemental_material/results/CaCoFold-R3D/CaCoFold-R3D_Rfam_vshuffle/RF00026_U6.R2R.sto.pdf]

RF00026_U6

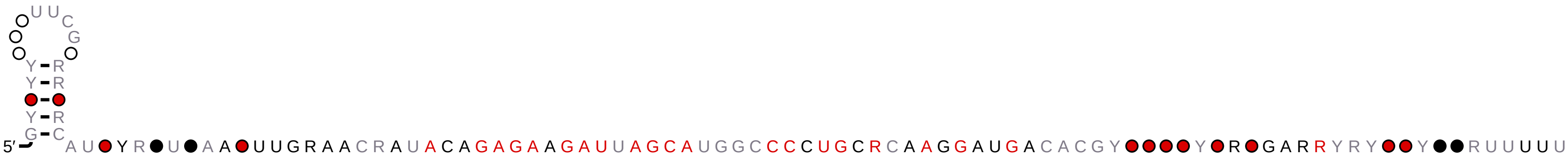
5′
A U Y R U A A U U G R A A C R A U A C A G A G A A G A U U A G C A U G G C C C U G C R C A A G G A U G A C A C G Y Y R G A R R Y R Y Y R U U U U U
Y Y Y G U U C G R R R C
